# Autophagy, TGF-β, and SMAD-2/3 Signaling Regulates Interferon-β Response in Respiratory Syncytial Virus Infected Macrophages

**DOI:** 10.3389/fcimb.2016.00174

**Published:** 2016-12-12

**Authors:** Swechha M. Pokharel, Niraj K. Shil, Santanu Bose

**Affiliations:** Department of Veterinary Microbiology and Pathology, Washington State UniversityPullman, WA, USA

**Keywords:** respiratory syncytial virus, autophagy, TGF-β, SMAD, macrophages, interferon-β

## Abstract

Human respiratory syncytial virus (RSV) is a lung tropic virus causing severe airway diseases including bronchiolitis and pneumonia among infants, children, and immuno-compromised individuals. RSV triggers transforming growth factor-β (TGF-β) production from lung epithelial cells and TGF-β facilitates RSV infection of these cells. However, it is still unknown whether RSV infected myeloid cells like macrophages produce TGF-β and the role of TGF-β if any during RSV infection of these cells. Our study revealed that RSV infected macrophages produce TGF-β and as a consequence these cells activate TGF-β dependent SMAD-2/3 signaling pathway. Further mechanistic studies illustrated a role of autophagy in triggering TGF-β production from RSV infected macrophages. In an effort to elucidate the role of TGF-β and SMAD-2/3 signaling during RSV infection, we surprisingly unfolded the requirement of TGF-β—SMAD2/3 signaling in conferring optimal innate immune antiviral response during RSV infection of macrophages. Type-I interferon (e.g., interferon-β or IFN-β) is a critical host factor regulating innate immune antiviral response during RSV infection. Our study revealed that loss of TGF-β—SMAD2/3 signaling pathway in RSV infected macrophages led to diminished expression and production of IFN-β. Inhibiting autophagy in RSV infected macrophages also resulted in reduced production of IFN-β. Thus, our studies have unfolded the requirement of autophagy—TGF-β—SMAD2/3 signaling network for optimal innate immune antiviral response during RSV infection of macrophages.

## Introduction

Human respiratory syncytial virus (RSV) causes severe lung diseases bronchiolitis and pneumonia among high risk individuals (e.g., infants, children, immuno-compromised individuals; Hall, 2001; Falsey et al., 2005). Innate immune antiviral response mediated by type-I interferon (e.g., interferon-β or IFN-β) is critical for combating virus infection (Uematsu and Akira, 2007; Wilkins and Gale, 2010; Newton and Dixit, 2012). IFN-β plays a pivotal role in host defense against RSV infection and is a major player driving virus clearance from the respiratory tract. RSV infected cells utilize various mechanisms including activation of pattern recognition receptors (PRRs), to trigger IFN-β release during infection (Sabbah et al., 2009; Tsai et al., 2015). In the current study we have unfolded a previously unknown mechanism regulating IFN-β production during RSV infection. We have identified a signaling network comprising of autophagy, transforming growth factor-β (TGF-β) and TGF-β induced SMAD-2/3 signaling in promoting IFN-β response in RSV infected macrophages.

Our current study stemmed from our attempt to understand the role of TGF-β in RSV infected macrophages. Previous studies have demonstrated TGF-β production from RSV infected lung epithelial cells (McCann and Imani, 2007; Gibbs et al., 2009; Mgbemena et al., 2011; Bakre et al., 2015). Epithelial cell specific TGF-β facilitated RSV infection of lung epithelial cells (McCann and Imani, 2007; Gibbs et al., 2009; Mgbemena et al., 2011). In addition, TGF-β regulated immune response in RSV infected neonatal dendritic cell-T cell co-culture (Thornburg et al., 2010). However, so far no studies have focused on the role of TGF-β induced SMAD-2/3 signaling during RSV infection. Recent studies have unfolded a “pivotal” role of myeloid cells like macrophages in regulating innate immunity and virus pathogenesis during respiratory virus infection (Peschke et al., 1993; Gordon and Read, 2002; Schneberger et al., 2011; Gwyer Findlay and Hussell, 2012; Aggarwal et al., 2014). In that regard, several aspect of TGF-β's function during RSV infection has yet to be investigated. It is unknown whether—(a) RSV infected macrophages produce TGF-β; (b) TGF-β activates SMAD-2/3 signaling during RSV infection of macrophages; (c) TGF-β and SMAD-2/3 signaling have any role in RSV infected macrophages; and (d) TGF-β and SMAD-2/3 signaling regulate antiviral signaling during RSV infection.

In the current study we have characterized the role of TGF-β and SMAD-2/3 signaling during RSV infection of macrophages. RSV triggered TGF-β production from macrophages, which resulted in activation of SMAD-2/3 signaling pathway. Surprisingly, induction of autophagy was required for SMAD-2/3 signaling during RSV infection. Furthermore, TGF-β and SMAD-2/3 signaling regulated antiviral response during RSV infection of macrophages since interferon-β (IFN-β) production was significantly reduced in cells lacking TGF-β dependent SMAD-2/3 signaling. Thus, our studies have demonstrated a role of autophagy—TGF-β—SMAD2/3 signaling network in positively regulating antiviral response (i.e., IFN-β production) during RSV infection.

## Results

### RSV triggers TGF-β production and activates SMAD-2/3 signaling in macrophages

**Figure 1:**
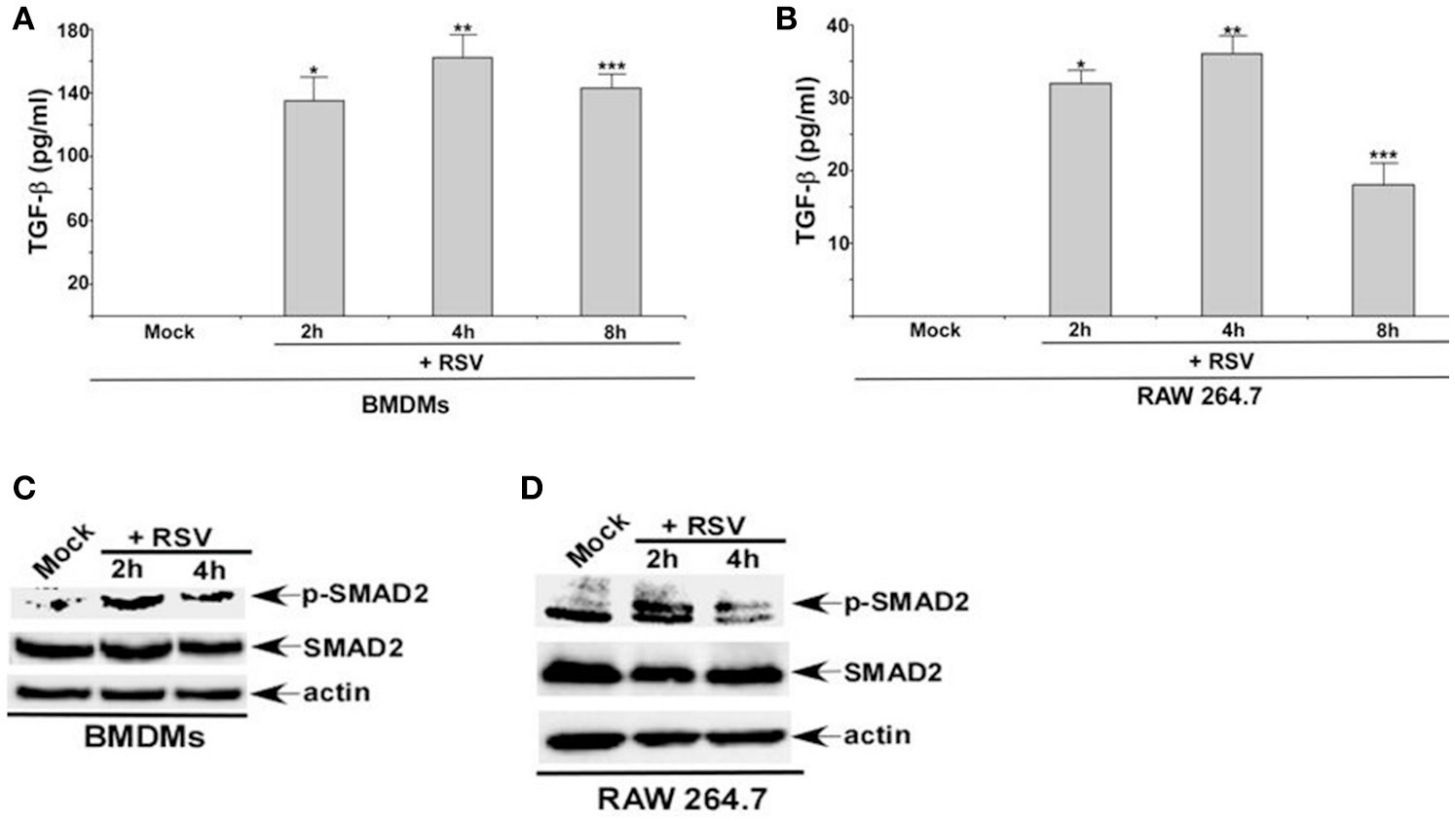
**RSV triggers TGF-β production and activates SMAD-2/3 signaling in macrophages. (A)** Primary bone marrow derived macrophages (BMDMs) and **(B)** mouse macrophage cell line RAW 264.7 were infected with RSV (1MOI) for different time points. Following infection, the medium supernatant was collected to assess active TGF-β secretion by ELISA analysis. **(C)** BMDMs and **(D)** RAW 264.7 cells were infected with RSV (1MOI) for 2 and 4 h. Cell lysates were subjected to western blot analysis with phospho-SMAD2 (p-SMAD2), SMAD2, and β-actin antibodies. The ELISA values **(A,B)** represent the mean ± standard deviation. ^*^p, ^**^p, and ^***^p ≤ 0.05 using a Student's *t*-test.

RSV infection results in TGF-β production from non-myeloid cells like lung epithelial cells (McCann and Imani, 2007; Gibbs et al., 2009; Mgbemena et al., 2011; Bakre et al., 2015). However, it is still unknown whether TGF-β is released from RSV infected myeloid cells like macrophages. Therefore, we investigated TGF-β production from RSV infected macrophages. For these studies, we infected primary mouse bone marrow derived macrophages (BMDMs) and mouse macrophage RAW 264.7 cell-line with RSV. At 2, 4, and 8 h post-infection, medium supernatant was collected to analyze active TGF-β levels by ELISA. RSV infection triggered TGF-β release from macrophages, since we detected TGF-β in the medium supernatant of RSV infected BMDMs and RAW 264.7 cells (Figures 1A,B). TGF-β production was independent of cell toxicity or cell death since we failed to detect LDH (LDH cytotoxicity assay), apoptosis and necrosis during the 2–8 h post-RSV infection (data not shown).

TGF-β activates SMAD-2/3 signaling pathway following binding to TGF-β receptor (TGF-βR) (Heldin and Moustakas, 2016). Although TGF-β production from RSV infected lung epithelial cells has been noted earlier (McCann and Imani, 2007; Gibbs et al., 2009; Mgbemena et al., 2011; Brubaker et al., 2015), it is still unknown whether RSV activates SMAD-2/3 signaling pathway. In order to investigate the status of SMAD-2/3 signaling during RSV infection, BMDMs, and RAW 264.7 cells were infected with RSV. Cell lysate derived from infected cells were subjected to Western blot analysis to detect phosphorylated form of SMAD-2 (phospho-SMAD2). SMAD-2 phosphorylation only occurs following TGF-β/TGF-βR mediated activation of SMAD-2/3 signaling. RSV infection resulted in activation of SMAD-2/3 signaling in macrophages. Phospho-SMAD2 was detected in RSV infected macrophages (Figures 1C,D). Thus, our study shows TGF-β production from RSV infected macrophages and subsequent activation of SMAD-2/3 signaling pathway in infected cells.

### Autophagy induction during RSV infection promotes TGF-β production and SMAD-2/3 signaling pathway activation

Although TGF-β is released from RSV infected epithelial cells (McCann and Imani, 2007; Gibbs et al., 2009; Mgbemena et al., 2011; Bakre et al., 2015), the mechanism triggering TGF-β production during infection is still unknown. Since RSV induces autophagy (Morris et al., 2011; Reed et al., 2013; Owczarczyk et al., 2015), we next examined a possible role of autophagy in positively regulating TGF-β production during RSV infection.

**Figure 2:**
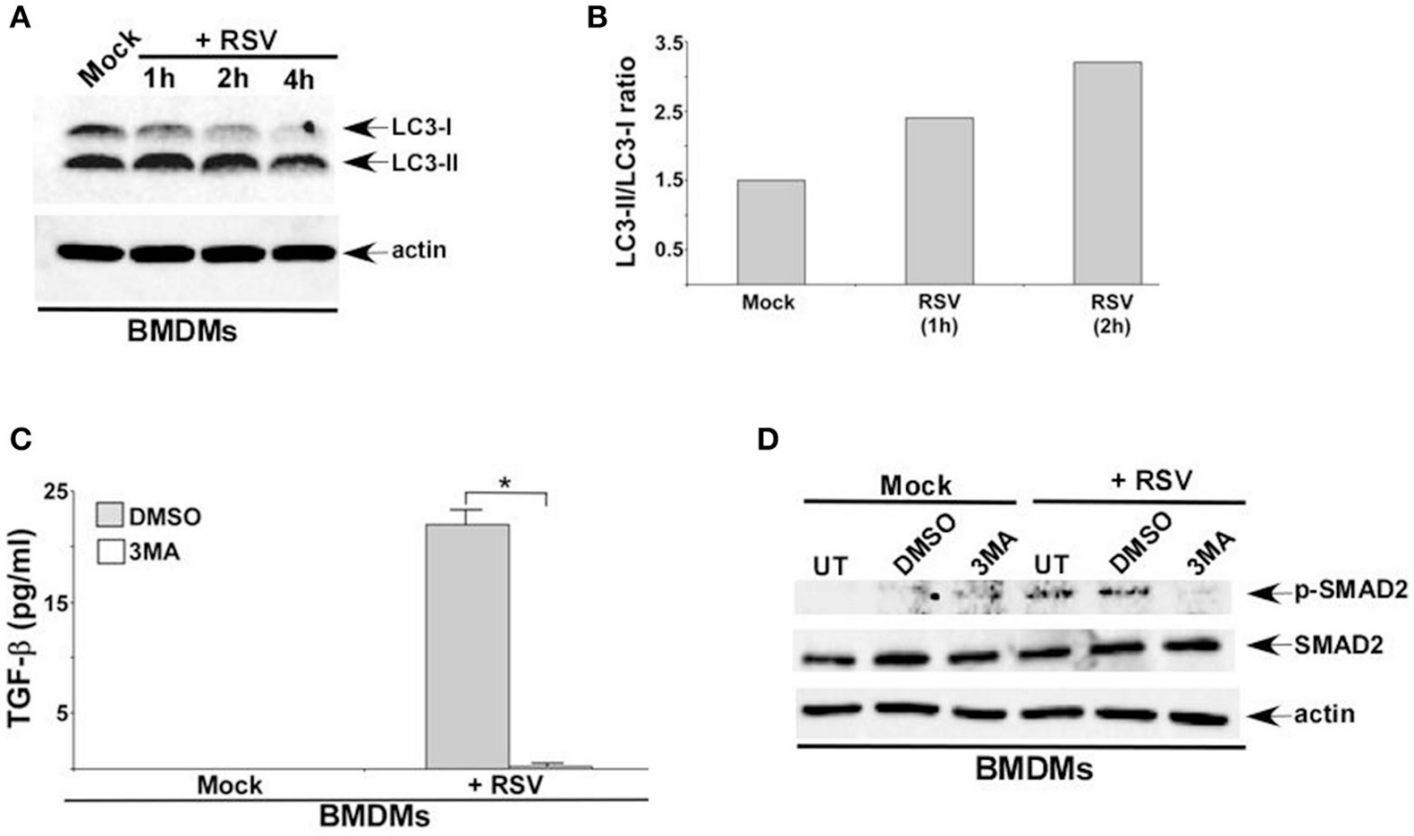
**Autophagy induction during RSV infection promotes TGF-β production and SMAD-2/3 signaling pathway activation. (A)** BMDMs were infected with RSV (1MOI) for 1, 2, and 4 h. Cell lysates from mock and RSV infected cells were subjected to western blotting with LC3 antibody. **(B)** Band intensity of LC3-I and LC3-II (Figure 2A) was quantified and the ratio of LC3-II/LC3-I was plotted to denote autophagy induction during RSV infection. **(C)** BMDMs were pre-treated with autophagy inhibitor (3 MA; 5 mM) for 2 h and infected with RSV (1MOI) in presence of DMSO (vehicle control) or 3 MA. Following infection, TGF-β levels in the medium supernatant was measured by ELISA. **(D)** BMDMs were pre-treated with 3 MA (5 mM) for 2 h and infected with RSV (1MOI) in presence of DMSO or 3 MA. Cell lysates were subjected to western blot analysis with phospho-SMAD2 (p-SMAD2), SMAD2, and β-actin antibodies. The ELISA value **(C)** represents the mean ± standard deviation. ^*^p ≤ 0.05 using a Student's *t*-test.

RSV induced autophagy in macrophages was deduced from Western blotting with LC3 antibody (Figures 2A,B). In order to assess autophagy's role, we treated cells with autophagy inhibitor 3 MA. Autophagy plays an important role in TGF-β production during RSV infection, since 3 MA treatment abrogated TGF-β release from RSV infected BMDMs (Figure 2C). A similar result was observed following treatment of RSV infected RAW 264.7 cells with 3 MA (data not shown). Autophagy was also required for activation of SMAD-2/3 signaling during RSV infection. Western blot analysis revealed drastic reduction in phospho-SMAD2 levels following treatment of RSV infected BMDMs with 3 MA (Figure 2D). A similar result was observed in 3 MA treated RAW 264.7 cells infected with RSV (data not shown).

**Figure 3:**
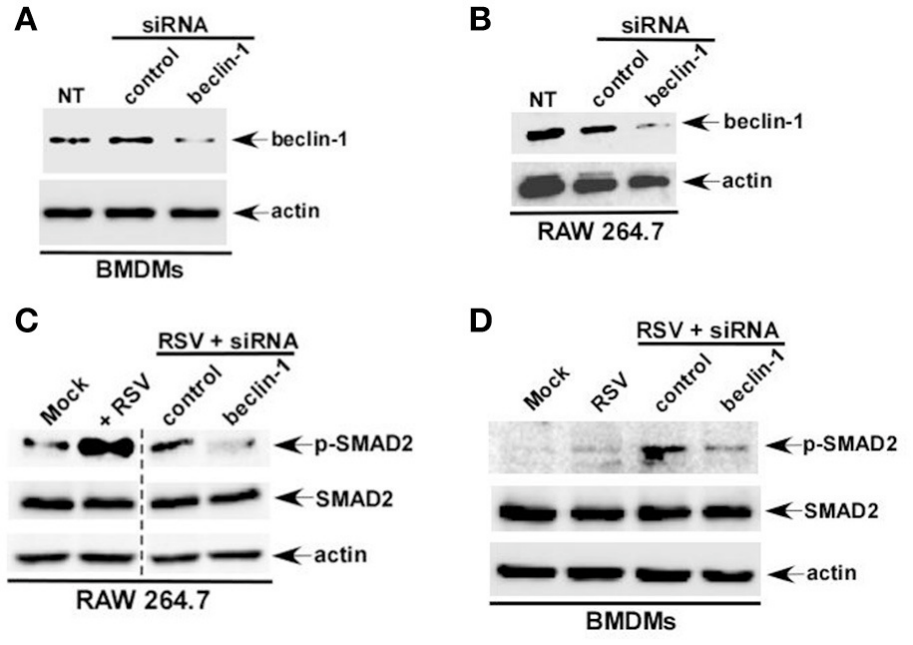
**Autophagy induction during RSV infection promotes SMAD-2/3 signaling pathway activation**. BMDMs **(A)** and RAW 264.7 cells **(B)** were transfected with either control siRNA or beclin-1 siRNA (60 pmol). Cell lysates collected from these cells were subjected to western blot analysis with beclin-1 and β-actin antibodies. RAW 264.7 cells **(C)** and BMDMs **(D)** cells silenced for beclin-1 expression were infected with RSV. Cell lysate collected from these cells were subjected to western blot analysis with phospho-SMAD2 (p-SMAD2), SMAD2, and β-actin antibodies. NT; non-transfected cells.

To further validate our results we generated macrophages deficient in beclin-1, a critical cellular factor required for autophagy induction. Beclin-1 silencing by siRNA led to loss of beclin-1 protein expression in BMDMs (Figure 3A) and RAW 264.7 (Figure 3B) cells. In accordance with our studies with 3 MA, we observed loss of phospho-SMAD2 protein following RSV infection of beclin-1 deficient BMDMs (Figure 3C) and RAW 264.7 (Figure 3D) macrophages. Thus, we have identified autophagy as one of the major cellular events triggering TGF-β production and SMAD-2/3 signaling pathway activation during RSV infection.

### TGF-β and SMAD-2/3 signaling is required for optimal interferon-β (IFN-β) production during RSV infection

Previous studies with lung epithelial cells revealed a role of TGF-β in modulating RSV infection (McCann and Imani, 2007; Gibbs et al., 2009; Mgbemena et al., 2011; Bakre et al., 2015), since blocking TGF-β activity reduced RSV infection of lung epithelial cells (McCann and Imani, 2007; Gibbs et al., 2009; Mgbemena et al., 2011). In that context, the exact role of TGF-β during RSV infection is still unknown. Moreover, studies exploring the role of SMAD-2/3 signaling during RSV infection are lacking.

**Figure 4:**
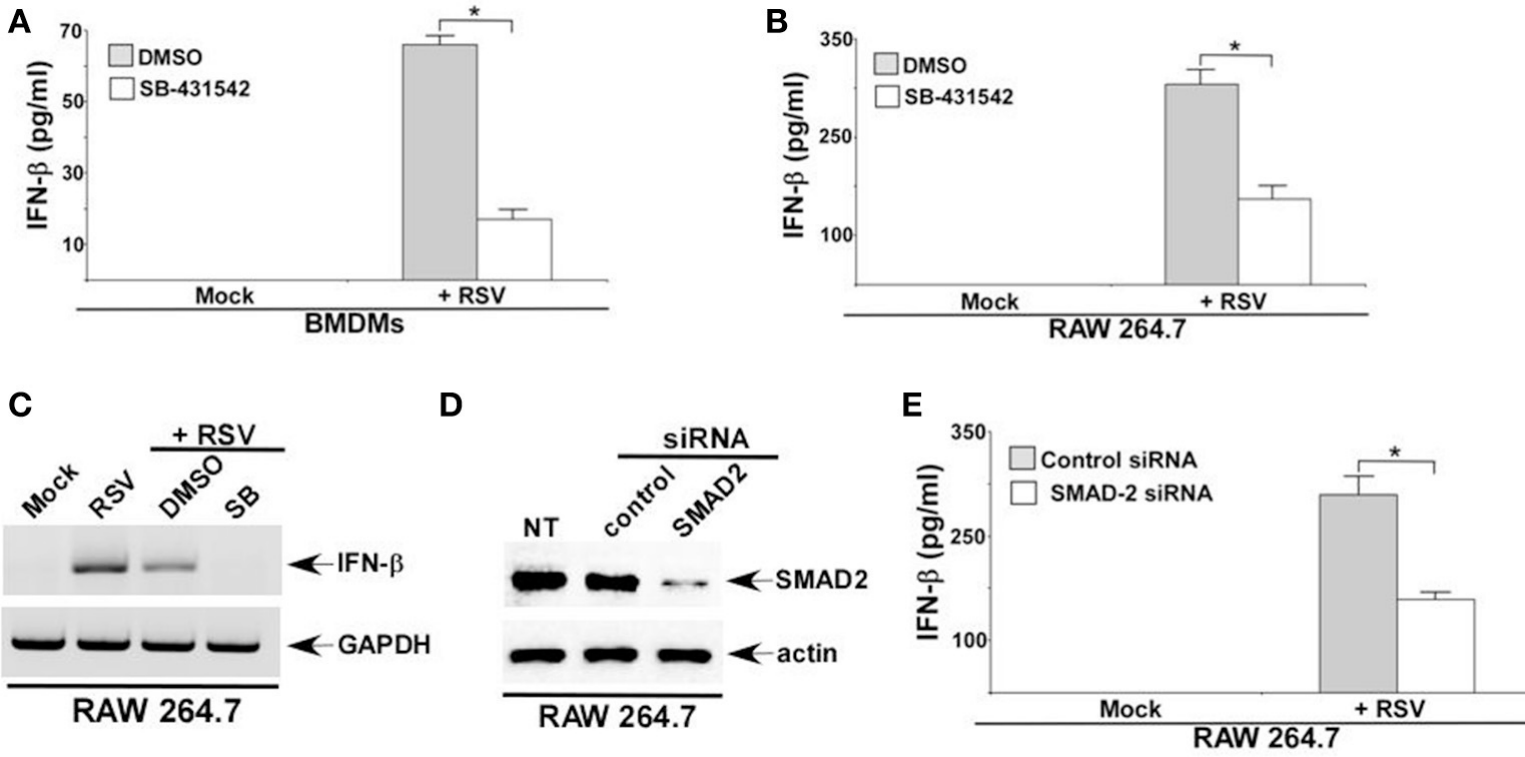
**TGF-β and SMAD-2/3 signaling is required for optimal interferon-β (IFN-β) production during RSV infection**. BMDMs **(A)** and RAW 264.7 cells **(B)** treated with either DMSO (vehicle control) or TGF-β inhibitor (SB-431542; 10 μM) were infected with RSV (1MOI; 16 h). Following infection, medium supernatant was assessed for IFN-β secretion by ELISA analysis. **(C)** RAW264.7 cells treated with either DMSO or SB-431542 (SB) were infected with RSV (1MOI). At 16 h post-infection, RT-PCR analysis was performed to assess IFN-β expression. **(D)** RAW 264.7 cells were transfected with either control siRNA or SMAD2 siRNA. Cell lysates collected from these cells were subjected to western blot analysis with SMAD2 and β-actin antibodies. **(E)** RAW 264.7 cells transfected with either control siRNA or SMAD2 siRNA were infected with RSV (1MOI). At 16 h post-infection, medium supernatant was collected to assess IFN-β level by ELISA. The ELISA values **(A,B,E)** represent the mean ± standard deviation. ^*^p ≤ 0.05 using a Student's *t*-test. NT; non-transfected cells.

RSV infection of macrophages is abortive (Segovia et al., 2012). Cellular entry and replication of RSV has been documented in macrophages. However, infectious RSV progeny virus is not released (virus budding) from macrophages. Although macrophages do not support RSV budding, RSV infected macrophages produce high level of IFN-β. It is now becoming apparent that type I interferon (IFN-α/β) released from RSV infected myeloid cells like macrophages are critical for airway host defense against RSV. Interestingly, TGF-β and SMAD-2/3 signaling can positively regulate type-I interferon production and response (Qing et al., 2004). In light of the role of TGF-β and SMAD-2/3 signaling in modulating IFN-β response, we next investigated whether TGF-β—SMAD2/3 pathway regulates IFN-β production during RSV infection. For these studies we utilized the TGF-β inhibitor SB-431542, that blocks extracellular TGF-β activity by inhibiting TGF-β binding to TGF-β receptor. In addition, we utilized SMAD-2 deficient macrophages to study the role of SMAD-2/3 signaling during IFN-β response. TGF-β was required for optimal IFN-β response during RSV infection since significant reduction in IFN-β production was observed following treatment of RSV infected BMDMs (Figure 4A) and RAW 264.7 (Figure 4B) cells with SB-431542. SB-431542 treatment also drastically abrogated IFN-β expression in RSV infected macrophages (Figure 4C). These results were further validated by using SMAD-2 deficient macrophages. We used siRNA to silence SMAD-2 expression in RAW 264.7 cells. Reduced SMAD-2 protein expression was noted in cells transfected with SMAD-2 siRNA (Figure 4D). RSV infection of SMAD-2 deficient cells resulted in significant loss of IFN-β production (Figure 4E). These results have highlighted a mechanism of IFN-β production during RSV infection. Our study suggests a role of TGF-β induced SMAD-2/3 signaling pathway in promoting activation of antiviral response by virtue of facilitating production of IFN-β from RSV infected macrophages. These studies have also illustrated a biological role of TGF-β and SMAD-2/3 signaling in positively regulating antiviral response in macrophages during RSV infection.

**Figure 5:**
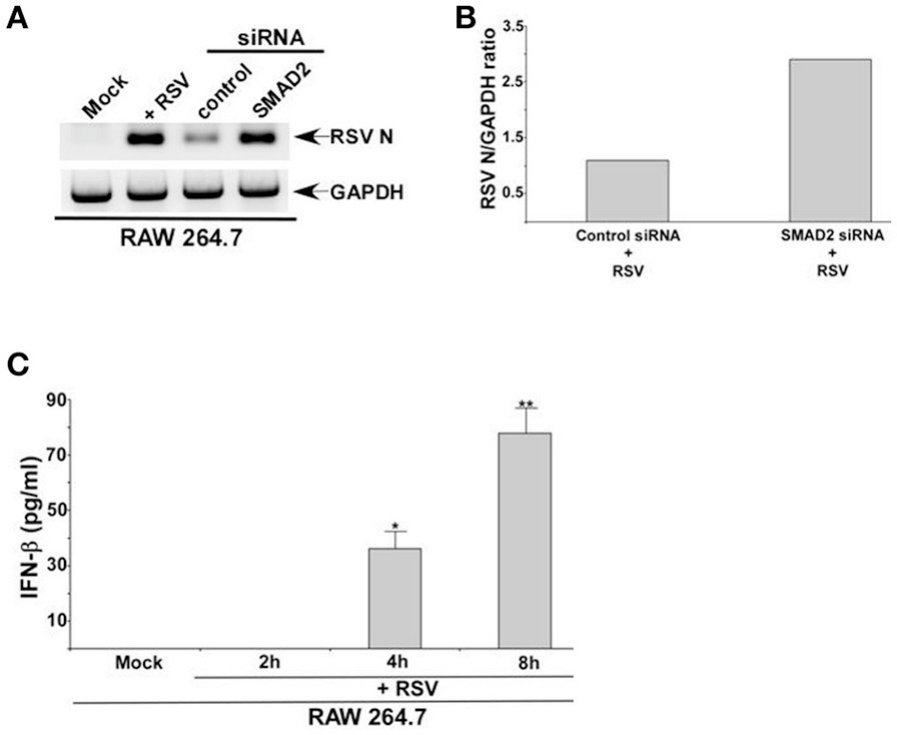
**TGF-β regulates RSV replication in macrophages and IFN-β production kinetics following RSV infection. (A)** RAW 264.7 cells transfected with either control siRNA or SMAD2 siRNA were infected with RSV (1MOI). At 16 h post-infection, RT-PCR analysis was performed to assess expression RSV nucleocapsid (N) protein mRNA. **(B)** Band intensity of RSV N and GAPDH in control siRNA and SMAD2 siRNA transfected cells (Figure 5A) was quantified and the ratio of N/GAPDH was plotted to denote enhanced expression of RSV N in SMAD2 siRNA transfected cells. **(C)** RAW 264.7 were infected with RSV (1MOI) for 0 h (mock), 2, 4, and 8 h. Following infection, the medium supernatant was collected to assess IFN-β production by ELISA analysis. The ELISA value represents the mean ± standard deviation. ^*^p and ^**^p ≤ 0.05 using a Student's *t*-test.

The role of TGF-β and SMAD-2/3 signaling in positively regulating IFN-β production was validated by investigating RSV infection in macrophages. IFN-β mediated antiviral response restricts RSV replication and therefore, loss of IFN-β production results in enhanced viral replication. Since RSV does not productively infect macrophages, we did not perform plaque assay analysis with medium supernatant of RSV infected macrophages. Instead, we analyzed RSV replication by evaluating expression of RSV nucleocapsid (N) protein mRNA. Indeed diminished IFN-β production from TGF-β signaling inhibited cells led to enhanced RSV replication since elevated expression of RSV N mRNA was observed in cells silenced for SMAD2 expression (Figures 5A,B). Similar enhanced expression of RSV fusion (F) protein was noted following Western blot analysis (with RSV F protein antibody) of RSV infected cell lysate derived from macrophages treated with TGF-β inhibitor SB-431542 (data not shown). A temporal relationship of TGF-β and IFN-β production during RSV infection was also evident. Although we detected TGF-β production from RSV infected macrophages at 2 h post-infection (Figures 1A,B), we failed to detect IFN-β at 2 h post-infection (Figure 5C). However, IFN-β could be detected at 4 h post-RSV infection (Figure 5C). This result demonstrated TGF-β production preceding IFN-β production during RSV infection and therefore this temporal event suggested TGF-β mediated regulation of IFN-β production.

### Autophagy regulates IFN-β production from RSV infected macrophages

**Figure 6:**
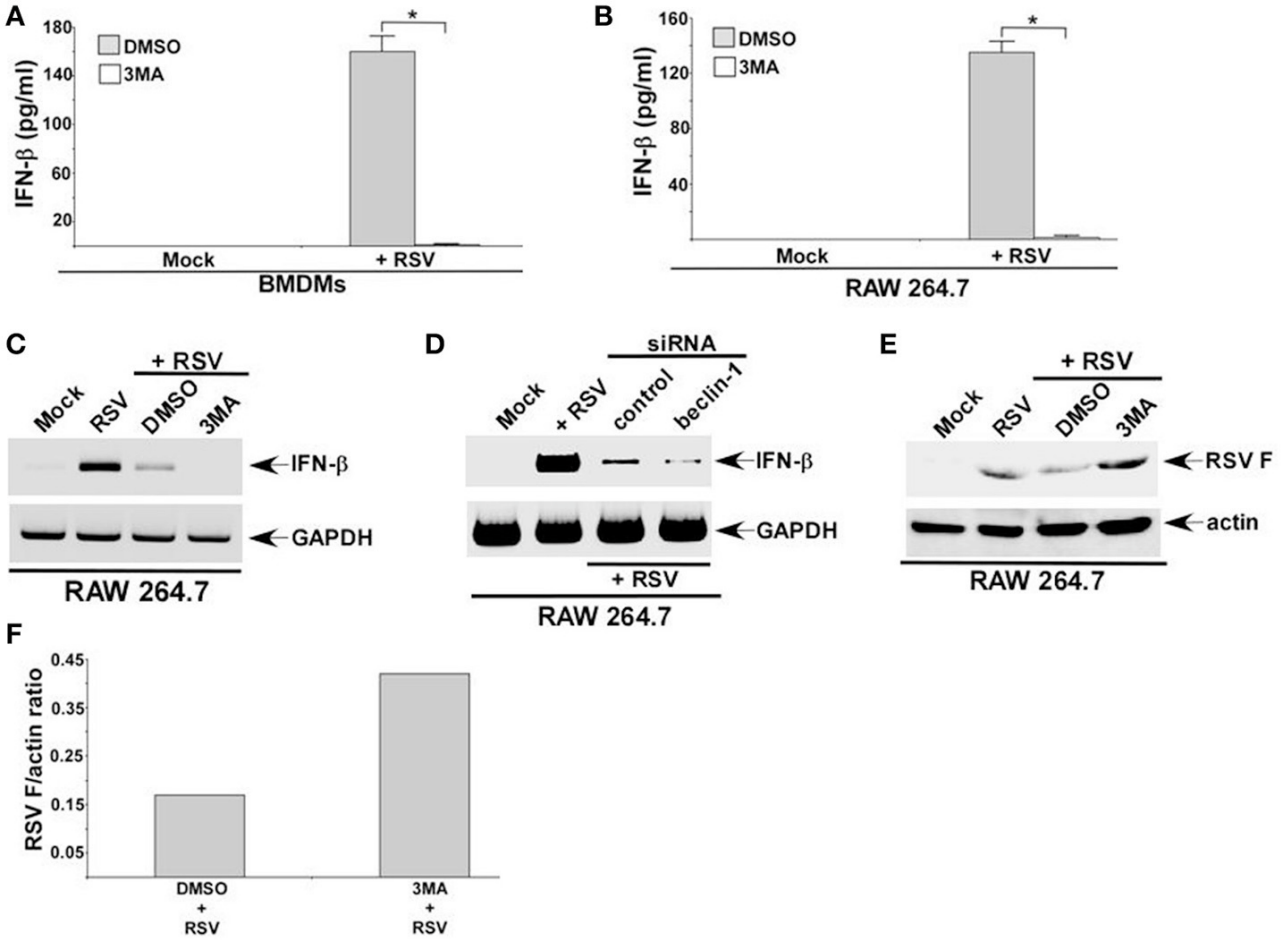
**Autophagy regulates IFN-β production from RSV infected macrophages**. BMDMs **(A)** and RAW 264.7 cells **(B)** treated with either DMSO (vehicle control) or autophagy inhibitor (3 MA; 5 mM) were infected with RSV (1MOI). At 16 h post-RSV infection, medium supernatant was collected to assess IFN-β production by ELISA. **(C)** RAW 264.7 cells treated with either DMSO or 3 MA were infected with RSV (1MOI). At 16 h post-infection, RT-PCR analysis was performed to analyze IFN-β expression. **(D)** RAW 264.7 cells transfected with either control siRNA or beclin-1 siRNA were infected with RSV (1MOI). At 16 h post-infection, RT-PCR analysis was performed to analyze IFN-β expression. **(E)** RAW 264.7 cells treated with either DMSO (vehicle control) or autophagy inhibitor (3 MA; 5 mM) were infected with RSV (1MOI). At 16 h post-RSV infection, cell lysate was subjected to Western blot analysis with RSV fusion **(F)** protein antibody. **(F)** Band intensity of RSV F and actin in DMSO and 3 MA treated cells (Figure 6E) was quantified and the ratio of F protein/actin was plotted to denote enhanced expression of RSV F protein in 3 MA treated cells. The ELISA values **(A,B)** represent the mean ± standard deviation. ^*^p ≤ 0.05 using a Student's *t*-test.

Autophagy triggered TGF-β—SMAD2/3 signaling, which was involved in IFN-β production during RSV infection of macrophages. These results suggested a role of autophagy in IFN-β production from infected macrophages. Interestingly, autophagy induction was required for optimal IFN-β production from RSV infected dendritic cells (DCs) and mouse airway (Morris et al., 2011; Reed et al., 2013; Owczarczyk et al., 2015). However, autophagy induction has not been studied yet in RSV infected macrophages. RSV infection led to autophagy induction in primary BMDMs (Figures 2A,B) and macrophage cell-line RAW 264.7 cells (data not shown). Role of autophagy was next evaluated by analyzing IFN-β production from macrophages treated with autophagy inhibitor 3 MA. Autophagy induction in macrophages during RSV infection was required for IFN-β production since 3 MA treatment drastically reduced IFN-β release from RSV infected BMDMs (Figure 6A) and RAW 264.7 (Figure 6B) macrophages. Concomitantly, RSV failed to induce IFN-β expression in 3 MA treated macrophages (Figure 6C). This result was further validated by using autophagy deficient RAW 264.7 cells that are silenced for beclin-1 expression (Figure 3B). RSV infection of beclin-1 deficient macrophages led to drastic reduction in IFN-β expression (Figure 6D). Loss of IFN-β production from autophagy inhibited cells led to enhanced RSV replication since elevated RSV F protein expression was observed in cells treated with autophagy inhibitor 3 MA (Figures 6E,F). These results have demonstrated a critical role of autophagy in triggering antiviral response in RSV infected macrophages by promoting IFN-β release.

## Discussion

**Figure 7:**
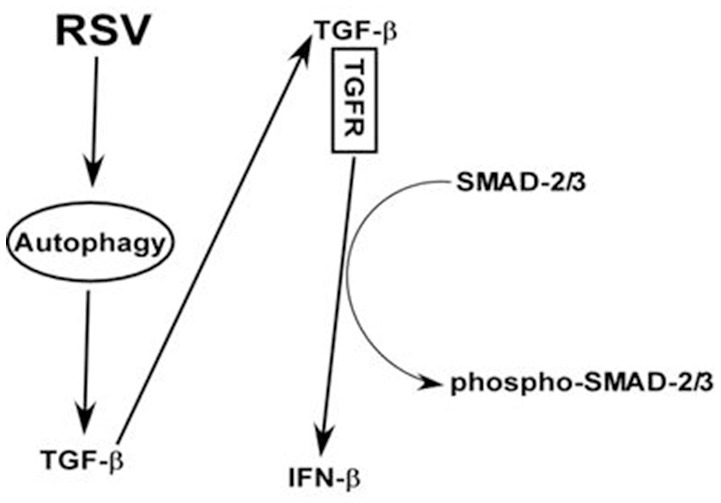
**A model depicting the role of autophagy—TGF-β—SMAD2/3 signaling network in positively regulating IFN-β production during RSV infection of macrophages**. RSV infection triggers autophagy in macrophages and this event promotes TGF-β production. Cell surface TGF-β receptor (TGFR) is then activated by extracellular TGF-β via paracrine/autocrine action. Interaction of extracellular TGF-β with TGFR will result in activation of SMAD-2/3 signaling comprising of phosphorylation of SMAD-2/3. Activation (i.e., phosphorylation of SMAD-2/3) of SMAD-2/3 will result in IFN-β expression and production.

Based on our study, we postulate a scheme (and a model) of IFN-β production from RSV infected macrophages (Figure 7). RSV infected macrophages will induce autophagy, which will trigger TGF-β release. Cell surface TGF-β receptor is then activated by extracellular TGF-β via paracrine/autocrine action. This will culminate in activation (i.e., phosphorylation of SMAD-2/3) of SMAD-2/3 pathway and subsequent expression/production of IFN-β. Thus, our studies have illustrated an important role of autophagy— TGF-β—SMAD2/3 pathway in launching an antiviral response (i.e., IFN-β production) in RSV infected macrophages.

TGF-β is a pleotropic cytokine regulating a plethora of biological functions and activities, including immune surveillance, pro-, and anti-inflammatory response, and cell-cycle regulation (Travis and Sheppard, 2014; Heldin and Moustakas, 2016). Respiratory RNA viruses like influenza A virus (IAV), RSV and rhinovirus triggers TGF-β expression and release from non-myeloid cells like lung epithelial cells and fibroblasts (Thomas et al., 2009; Bedke et al., 2012; Li et al., 2015). Surprisingly, we and others have shown a presumptive detrimental role of TGF-β in host defense against respiratory RNA viruses. TGF-β facilitated RSV (McCann and Imani, 2007; Gibbs et al., 2009; Mgbemena et al., 2011) and rhinovirus (Thomas et al., 2009; Bedke et al., 2012) infection of lung epithelial cells and airway fibroblasts. Accordingly, TGF-β diminished antiviral response by reducing type-I IFN production from rhinovirus infected fibroblasts and epithelial cells (Thomas et al., 2009; Bedke et al., 2012). An interesting aspect of TGF-β is its cell-type dependent response. Particularly, TGF-β activity (and response) may profoundly differ in myeloid vs. non-myeloid cells. In that context, there is only one study showing a respiratory virus (RSV) up-regulating TGF-β in a myeloid cell (cord blood DCs) and studies with DC-T cell co-culture (RSV infected DC co-cultured with T-cells) revealed a role of TGF-β in modulating *in vitro* T-cell response (Thornburg et al., 2010). Due to limited studies with myeloid cells, particularly with no studies being performed with macrophages, we investigated whether—(a) RSV triggers TGF-β release from macrophages; and (b) TGF-β produced from RSV infected macrophages plays any functional role in regulating innate immune response. Our studies have demonstrated that—(a) TGF-β is released from RSV infected macrophages; and (b) TGF-β—SMAD2/3 signaling is required for optimal IFN-β production during RSV infection.

SMAD-2/3 pathway represents the major TGF-β signaling cascade responsible for transmitting intracellular response originating on the cell surface following interaction of TGF-β with type-II TGF-β receptor (Heldin and Moustakas, 2016). So far no studies have focused on the SMAD-2/3 pathway during respiratory virus infection. It is unknown whether—(a) respiratory viruses like RSV activates SMAD-2/3 pathway; and (b) SMAD-2/3 pathway play any role in regulating virus infection and innate immune response. Our study revealed —(a) activation of SMAD-2/3 pathway in RSV infected macrophages; and (b) a role of SMAD-2/3 pathway in triggering IFN-β production during RSV infection and thus, “positively” regulating innate antiviral response.

Interferon regulatory factors (IRFs like IRF3, IRF7) play pivotal role in antiviral response (Stark et al., 1998; Honda et al., 2005; Ciancanelli et al., 2015). IRF3 and IRF7 are transcription factors residing in the cytoplasm of resting cells. They are activated (phosphorylated) by upstream signaling cascade originating from activated PRRs like toll-like receptors (TLRs) (Uematsu and Akira, 2007; Wilkins and Gale, 2010; Newton and Dixit, 2012). Activated IRF3 and IRF7 translocate to the nucleus to transactivate IFN-α and IFN-β gene expression. TLR3 activation in macrophages during RSV infection (Tsai et al., 2015) culminates in IFN-β expression/production, by virtue of IRF3 and IRF7 activation (Casola et al., 2001; Jewell et al., 2007; Sabbah et al., 2009; Remot et al., 2016). In that regard, IRF7 is required for IFN-β gene expression following TLR3 activation (Siednienko et al., 2012). Interestingly, TGF-β and SMAD-2/3 signaling plays an important role in up-regulating IRF7 transcriptional activity (Qing et al., 2004). Mechanistically, IRF7 is complexed with activated SMAD-3 and this complex upon translocation to the nucleus co-operatively acts on the ISRE (Interferon Stimulated Response Element) to optimally express IFN-β gene. TGF-β signaling blockade diminished IRF7 dependent IFN-β expression and release. IRF7 is a virus specific IFN-β inducer operating during MyD88-independent TLR signaling (e.g., TLR3 signaling) (Honda et al., 2005). RSV induces IRF7 expression in cells and mice respiratory tract (Casola et al., 2001; Jewell et al., 2007; Remot et al., 2016). Furthermore, IRF7 is constitutively expressed (and induced following virus infection) in primary macrophages and macrophage cell-lines like RAW 264.7 cells (Wilden et al., 2009; Ning et al., 2011). In that scenario, we envision TGF-β released from RSV infected macrophages will activate cell surface TGF-β receptor. Subsequent activation of SMAD-2 and SMAD-3 will lead to translocation of SMAD-IRF7 complex to the nucleus to transactivate IFN-β gene expression. In the future we will conduct studies to elucidate the mechanism regulating TGF-β expression and release during RSV infection. Furthermore, we will investigate the role of IRF7 and SMAD-IRF7 complex in regulating IFN-β expression during RSV infection of macrophages.

Autophagy constitutes a critical cellular process that maintains cellular homeostasis for normal biological and physiological functions (Shibutani et al., 2015). RSV induces autophagy in dendritic cells (DCs) and in the respiratory tract of infected mice (Morris et al., 2011; Reed et al., 2013; Owczarczyk et al., 2015). Autophagy is required for efficient IFN-β response during RSV infection, since lack of autophagy reduces IFN-β release from RSV infected DCs. Although RSV induced autophagy in DCs, autophagy induction in RSV infected macrophages has not been reported yet. We now show autophagy induction in RSV infected macrophages. Furthermore, our studies have illustrated a functional role of autophagy in positively regulating antiviral response (IFN-β production) in macrophages infected with RSV. Given the fact that TLR3 induces autophagy in macrophages (Delgado et al., 2008) and TLR3 is activated by RSV in these cells (Tsai et al., 2015), we postulate that autophagy induction in RSV infected macrophages occur due to RSV mediated activation of TLR3 pathway. A relationship between autophagy and TLR3 is evident from strikingly similar lung cytokine profile and pathogenesis severity in RSV infected autophagy deficient (i.e., beclin-1 deficient mice) and TLR3 knockout mice (Reed et al., 2013). In addition to TLR3, it is plausible that ssRNA genome of RSV can induce autophagy following TLR7 activation. TLR7 activation results in autophagy induction (Sanjuan et al., 2007; Delgado et al., 2008) and thus, during early infection ssRNA originating from RSV can induce autophagy via TLR7.

In summary, our studies have resulted in identification of autophagy—TGF-β–SMAD2/3 signaling network in macrophages as a critical cellular event required for optimal antiviral response (IFN-β production) following RSV infection.

## Materials and methods

### Virus and cell culture

Human respiratory syncytial virus (RSV A2 strain) was propagated in CV-1 cells and purified by centrifuging two times on discontinuous sucrose gradients as described previously (Segovia et al., 2012; Tsai et al., 2015). C57BL/6J mice were obtained from Jackson Laboratory. Bone marrow derived macrophages (BMDMs) were derived from femur and tibia of wild type (WT) mice and were cultured for 6–8 days as described earlier (Segovia et al., 2012; Tsai et al., 2014, 2015). Unless otherwise stated, BMDMs were plated on 12 well plates containing Roswell Park Memorial Institute medium 1640 (RPMI) (Gibco), 10% FBS (Gibco), 100 IU/mL penicillin, 100 mg/mL streptomycin (Gibco), and 20 ng/ml GM-CSF (Peprotech). RAW264.7 (mouse macrophage cell line) cells obtained from ATCC were maintained in Dulbecco's modified Eagle medium (DMEM), 10% FBS, 100 IU/mL penicillin, 100 mg/mL streptomycin unless otherwise noted.

Animal studies were performed according to housing and care of laboratory animals guidelines established by National Institutes for Health (NIH). All animal experiments were reviewed and approved by the Institutional Animal Care and Use Committee (IACUC) of Washington State University.

### Viral infection of cells

BMDMs and RAW264.7 cells were infected with purified RSV at one multiplicity of infection (MOI) in serum free antibiotic free OPTI-MEM medium (Gibco). Virus adsorption was performed for 1.5 h at 37°C. Following adsorption, cells were washed twice with DPBS (Dulbecco's phosphate-buffered saline) (Gibco) and the infection was continued in presence or absence of serum containing medium. To examine TGF-β secretion and SMAD-2 phosphorylation during RSV infection, BMDMs, and RAW264.7 cells were cultured in serum free medium.

In some experiments, cells were pre-treated with 3-methyladenine (3 MA; Sigma Aldrich) or TGF-β inhibitor (SB-431542; InVivogen). After pre-treatment, cells were infected with RSV and infection was continued in presence or absence of respective inhibitors. Dimethyl sulfoxide (DMSO) served as a vehicle control for SB-431542 and 3 MA.

### siRNA transfection

Mouse beclin-1 and mouse SMAD-2 were silenced in BMDMs or RAW 264.7 cells by using Lipofectamine 2000 for 24–48 h. Control siRNA, mouse beclin-1 siRNA and mouse SMAD-2 siRNA were purchased from Santa Cruz Biotechnology. Cells were transfected with 60 pmol of siRNAs.

### ELISA assay

Medium supernatants collected from RAW 264.7 cells and BMDMs were analyzed for active TGF-β (ebioscience) and IFN-β (PBL Assay Science) levels by cytokine-specific ELISA kit.

### RNA isolation and reverse transcriptase-PCR (RT-PCR)

Total RNA was extracted using TRIzol reagent (Life Technologies) following supplier's instructions. RT-PCR analysis was performed as described previously (Tsai et al., 2014, 2015; Segovia et al., 2015). MultiScribe reverse transcriptase (Applied Biosystem) was used to synthesize template cDNA. PCR was performed using 2X Taq Red master mix (Apex) in final reaction of 25 μl. The amplification cycle was as follows: an initial denaturing step (95°C for 5 min) was followed by 28–30 cycles of denaturing (95°C for 30 s), annealing (55–60°C for 30 s–1 min), and extending (72°C for 30 s), followed by either 5 or 10 min at 72°C for elongation. Following amplification, the PCR products were analyzed on 1% agarose gels and bands were visualized by ChemiDoc XRS (BioRad). Housekeeping gene glyceraldehyde-3-phosphate dehydrogenase (GAPDH) was used as a loading control. The primers used to detect the indicated gene are listed below:

Mouse GAPDH forward, (5′wordGCCAAGGTCATCCATGACAACTTTGG-3′) and mouse GAPDH reverse, (5′GCCTGCTTCACCACCTTCTTGATGTC-3′)
Mouse IFN-β forward, (5′-wordCAGCTCCAGCTCCAAGAAAGGACGAACATTCG-3′) and mouse IFN-β reverse, (5′-wordCCACCACTCATTCTGAGGCATCAACTGACAGG-3′)
RSV-N forward, (5′-wordGGAACAAGTTGTTGAGGTTTATGAATATGC-3′) and
RSV-N reverse, (5′-wordTTCTGCTGTCAAGTCTAGTACACTGTAGT-3′)

For some experiments, the PCR product bands were quantified using Image Lab Software (Bio-Rad).

### Western blotting

BMDMs and RAW264.7 cells were lysed using 1%-Triton X-100 in PBS (pH 7.4), EDTA-free protease inhibitor cocktail (Roche Diagnostics) and 10 mM of sodium pyrophosphate (Sigma) in PBS. Cell lysates were subjected to 10–15% SDS-PAGE. Separated proteins were transferred onto 0.2 μm nitrocellulose membrane (GE Health care) and blotted with specific antibodies. SMAD-2, phospho-SMAD2 and LC-3 antibodies were purchased from Cell signaling. Beclin-1 antibody was purchased from Abcam. RSV fusion (F) protein antibody was obtained from FDA (Dr. Judy Beeler). β-actin antibody was purchased from Bethyl Laboratories. For some experiments, protein bands from Western blot were quantified using Image Lab Software (Bio-Rad).

## Author contributions

SP, NS, and SB designed the experiments; SP and NS performed the experiments; SP, NS, and SB analyzed the data; SP and SB wrote the paper.

### Conflict of interest statement

The authors declare that the research was conducted in the absence of any commercial or financial relationships that could be construed as a potential conflict of interest.
